# Author Correction: Scutoids are a geometrical solution to three-dimensional packing of epithelia

**DOI:** 10.1038/s41467-018-06671-7

**Published:** 2018-10-08

**Authors:** Pedro Gómez-Gálvez, Pablo Vicente-Munuera, Antonio Tagua, Cristina Forja, Ana M. Castro, Marta Letrán, Andrea Valencia-Expósito, Clara Grima, Marina Bermúdez-Gallardo, Óscar Serrano-Pérez-Higueras, Florencia Cavodeassi, Sol Sotillos, María D. Martín-Bermudo, Alberto Márquez, Javier Buceta, Luis M. Escudero

**Affiliations:** 1 Departamento de Biología Celular, Universidad de Sevilla and Instituto de Biomedicina de Sevilla (IBiS), Hospital Universitario Virgen del Rocío/CSIC/Universidad de Sevilla, 41013 Seville, Spain; 2 CABD, CSIC/JA/UPO, Campus Universidad Pablo de Olavide, 41013 Seville, Spain; 3 Departamento de Matemática Aplicada I, Universidad de Sevilla, 41012 Seville, Spain; 4 Centro de Biología Molecular Severo Ochoa and CIBER de Enfermedades Raras, C/ Nicolás Cabrera 1, 28049 Madrid, Spain; 5 St. George’s, University of London, Cranmer Terrace, London, SW17 0RE UK; 6 Bioengineering Department, Lehigh University, Bethlehem, PA 18018 USA; 7 Chemical and Biomolecular Engineering Department, Lehigh University, Bethlehem, PA 18018 USA

Correction to: *Nature Communications* 10.1038/s41467-018-05376-1; published online: 27 July 2018

The original version of this article contained an error in ref. 39, which incorrectly cited ‘Fristrom, D. & Fristrom, J. W. in The Development of Drosophila melanogaster (eds. Bate, M. & Martinez-Arias, A.) II, (Cold spring harbor laboratory press, 1993)’. The correct reference is ‘Condic, M.L, Fristrom, D. & Fristrom, J.W. Apical cell shape changes during Drosophila imaginal leg disc elongation: a novel morphogenetic mechanism. *Development* **111**: 23–33 (1991)’. Furthermore, the last sentence of the fourth paragraph of the introduction incorrectly omitted citation of work by Rupprecht et al. The correct citation is given below. These errors have now been corrected in both the PDF and HTML version of this article.

Rupprecht, J.F., et al. Geometric constraints alter cell arrangements within curved epithelial tissues. *Mol. Biol. Cell* **28**, 3582–3594 (2017).

