# Supplementary material for: Scutoids are a geometrical solution to three-dimensional packing of epithelia
Source: Nat Commun. 2018 Jul 27;9:2960. doi: 10.1038/s41467-018-05376-1 (PMC6063940; doi:10.1038/s41467-018-05376-1)
Supplement: Supplementary file 1 — Supplementary Information [file 41467_2018_5376_MOESM1_ESM.pdf]

Supplementary Information:

Scutoids are a geometrical solution to three-dimensional packing of epithelia

Gomez-Galvez *et al.*

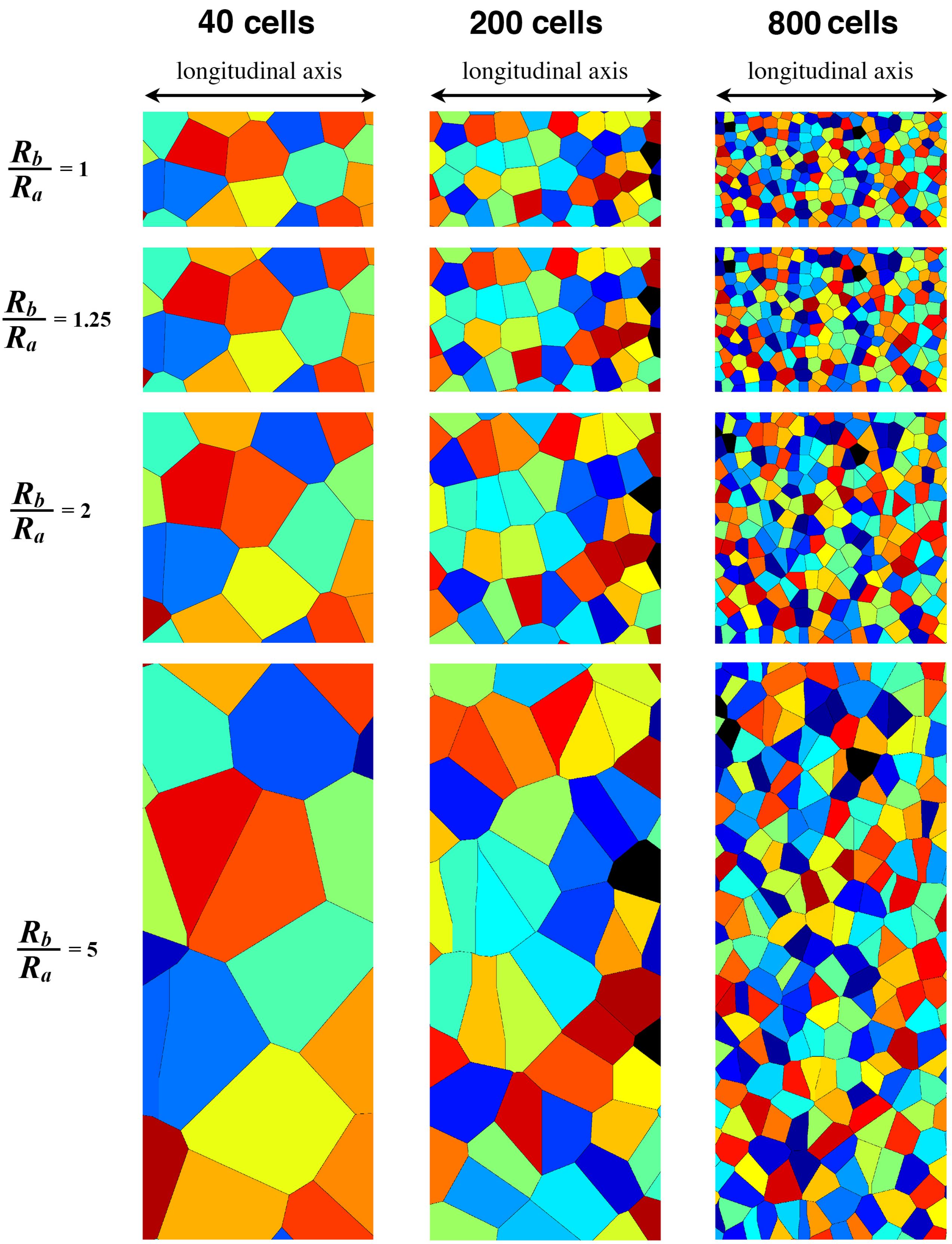

40 cells
200 cells
800 cells
longitudinal axis
longitudinal axis
longitudinal axis
$\frac{R_b}{R_a} = 1$
$\frac{R_b}{R_a} = 1.25$
$\frac{R_b}{R_a} = 2$
$\frac{R_b}{R_a} = 5$

Supplementary Figure 1. Design of the cylinder model. Different developed expanded basal surfaces of the mathematical model of tubular epithelia for the cases of 40, 200 and 800 cells. Every image represents 1/4 of the total cylinder length (along the longitudinal axis direction) and the full width. The cellular organization changes when the surface is expanded.

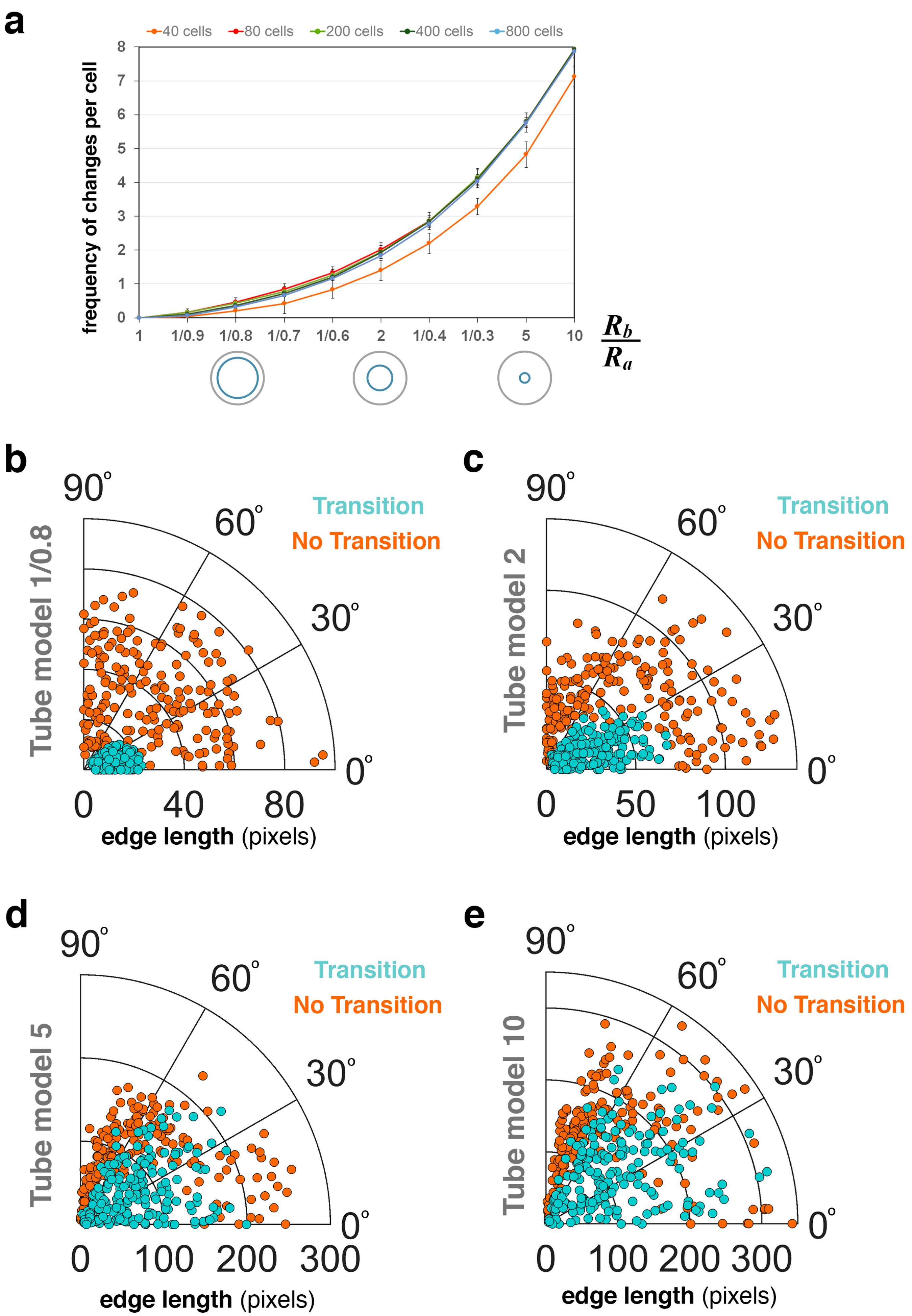

a
40 cells
80 cells
200 cells
400 cells
800 cells
frequency of changes per cell
0
1
2
3
4
5
6
7
8
1
1/0.9
1/0.8
1/0.7
1/0.6
2
1/0.4
1/0.3
5
10
$\frac{R_b}{R_a}$
b
Tube model 1/0.8
90°
60°
30°
0°
Transition
No Transition
0
40
80
edge length (pixels)
c
Tube model 2
90°
60°
30°
0°
Transition
No Transition
0
50
100
edge length (pixels)
d
Tube model 5
90°
60°
30°
0°
Transition
No Transition
0
100
200
300
edge length (pixels)
e
Tube model 10
90°
60°
30°
0°
Transition
No Transition
0
100
200
300
edge length (pixels)

Supplementary Figure 2. Apico-basal transitions appearance in the tubular Voronoi model. a) Frequency of neighbour exchanges in relation to the increase of the surface ratio for five conditions (40, 80, 200, 400 and 800 cells). **b-e)** Polar scatter plots representing the angle and length of the edge in four-cell motifs from the tubular Voronoi model with different surface ratios (1/0.8, 2, 5 and 10). Light blue points stand for motifs that exchange neighbours; orange points stand for motifs that do not show apico-basal intercalations.

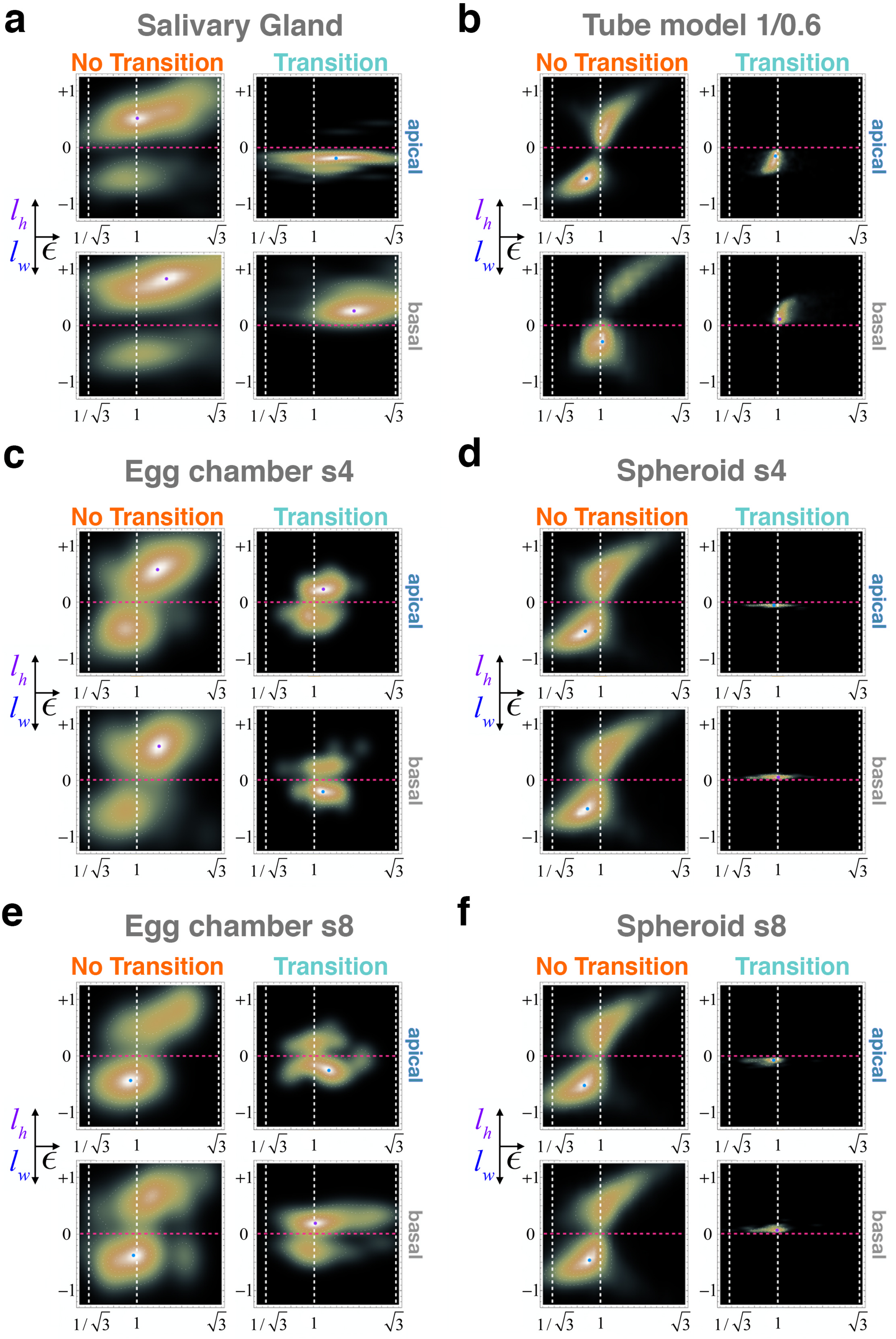

a
Salivary Gland
b
Tube model 1/0.6
c
Egg chamber s4
d
Spheroid s4
e
Egg chamber s8
f
Spheroid s8
No Transition
Transition
apical
basal
l_h
l_w
ϵ
+1
0
−1
$1/\sqrt{3}$
1
$\sqrt{3}$

Supplementary Figure 3. **Probability density of couples** (l, ϵ). The plots show the probability density of obtaining a couple (l, ϵ), categorized in terms of the configurations l_w and l_h (see Methods), as obtained from the analyses of data from Voronoi tubular (**b**) and spheroidal (**d**, **f**) models, salivary glands (**a**) and egg chambers (**c**, **e**). The dots in the panels indicate the absolute maximum of the probability density. Configurations close to the fourfold unstable configuration (magenta line), can undergo transitions driven by the surface ratio anisotropy (i.e. by changes in the cellular aspect ratio). The measured values of the aspect ratio statistically explained the results obtained about the most favorable, stable, energetic state in the cases of, both, transitions and no transitions in the context of the line-tension minimization model. In particular, if $\epsilon < 1$ the most favourable energetic state is l_w whereas if $\epsilon > 1$ then l_h is the preferred energetic state in agreement with the energetic model. In the case of transitions, the configurations close to the fourfold unstable configuration (magenta line), can undergo transitions driven by the surface ratio anisotropy (i.e. by changes in the cellular aspect ratio). The observed directionality of the transitions in Voronoi models (tubular and spheroidal) and the salivary gland agrees with the theoretical predictions favouring the l_w configuration in basal as the aspect ratio increases. In the case of the egg chambers (s4 and s8), the data show that the directionality of the transitions does not necessarily follow the energetic model and suggest the importance of active cellular/developmental processes.

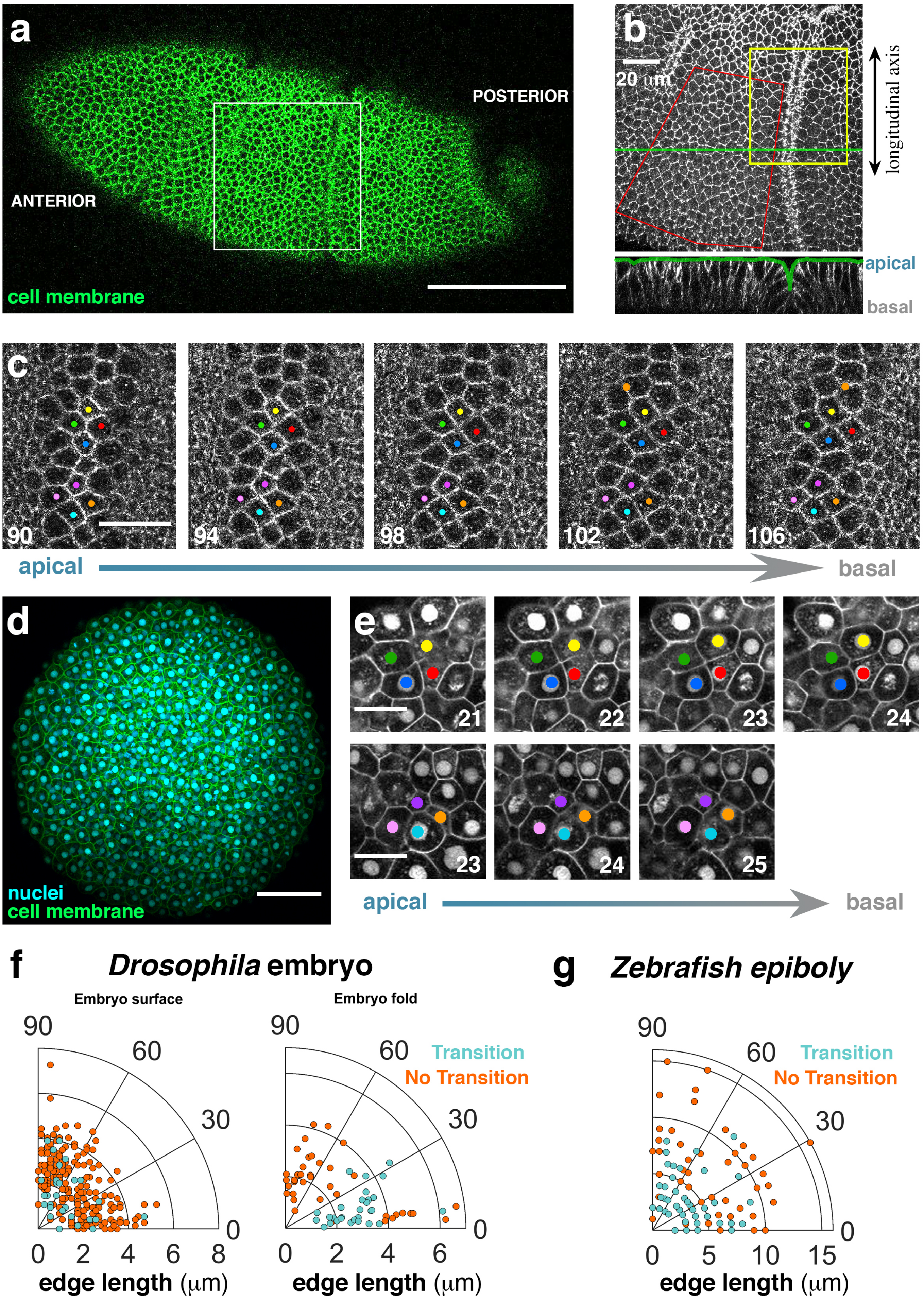

a
POSTERIOR
ANTERIOR
cell membrane
b
20 μm
longitudinal axis
apical
basal
c
90
94
98
102
106
apical
basal
d
nuclei
cell membrane
e
21
22
23
24
23
24
25
apical
basal
f
Drosophila embryo
Embryo surface
Embryo fold
Transition
No Transition
edge length (μm)
edge length (μm)
g
Zebrafish epiboly
Transition
No Transition
edge length (μm)

Supplementary Figure 4. Scutoids appear in different epithelial tissues. In each type of epithelia two transitions are shown. The dots label the cells participating in illustrative four-cells motifs (motif1: green, yellow, red and blue dots highlight cells in one motif; motif 2: pink, purple, orange and cyan dots highlight cells in another motif). **a)** Stage 6-7 *Drosophila* embryo showing the cells outlines and the anterior and posterior folds. Scale bar = 100 µm. **b)** Close up of the posterior region. The red line delimits a Region Of Interest (ROI) for the "areas without folds" where have been made the corresponding measurements. The yellow rectangle indicates a ROI in one of the gastrulation folds. The double arrow labels the direction of the longitudinal axis of the fold. The bottom part of the panel shows a sagittal section of the tissue along the green line in the upper part of the panel. The monolayer epithelium bends at the level of the gastrulation fold. Scale bar = 20 µm. **c)** Different confocal planes of the region inside of the yellow ROI in (**b**). The numbers indicate the confocal plane, increasing from apical to basal (outer to inner of the embryonic epithelium) regions of the cell. Every Z-section is separated by 0.3 µm. Scale bar = 20 µm. **d)** Confocal projection of a 50% epiboly zebrafish embryo with the cells membranes labelled in green and the nuclei in cyan. The outer surface of the zebrafish embryo corresponds with the apical region of the epithelial cells**.** Scale bar = 100 µm. **e)** High magnification image series showing the presence of apico-basal transitions in the outer layers of the embryonic epithelia. Every section of the stack is separated by 1.2 µm. Scale bar = 20 µm. **f-g)** Polar scatter plots illustrating the length and angle of the edge in four-cell motifs of the *Drosophila* embryo surface and fold (**f**), and in the 50% epiboly Zebrafish embryo (**g**), respectively. Light blue points stand for motifs that exchange neighbours; orange points stand for motifs that do not intercalate.

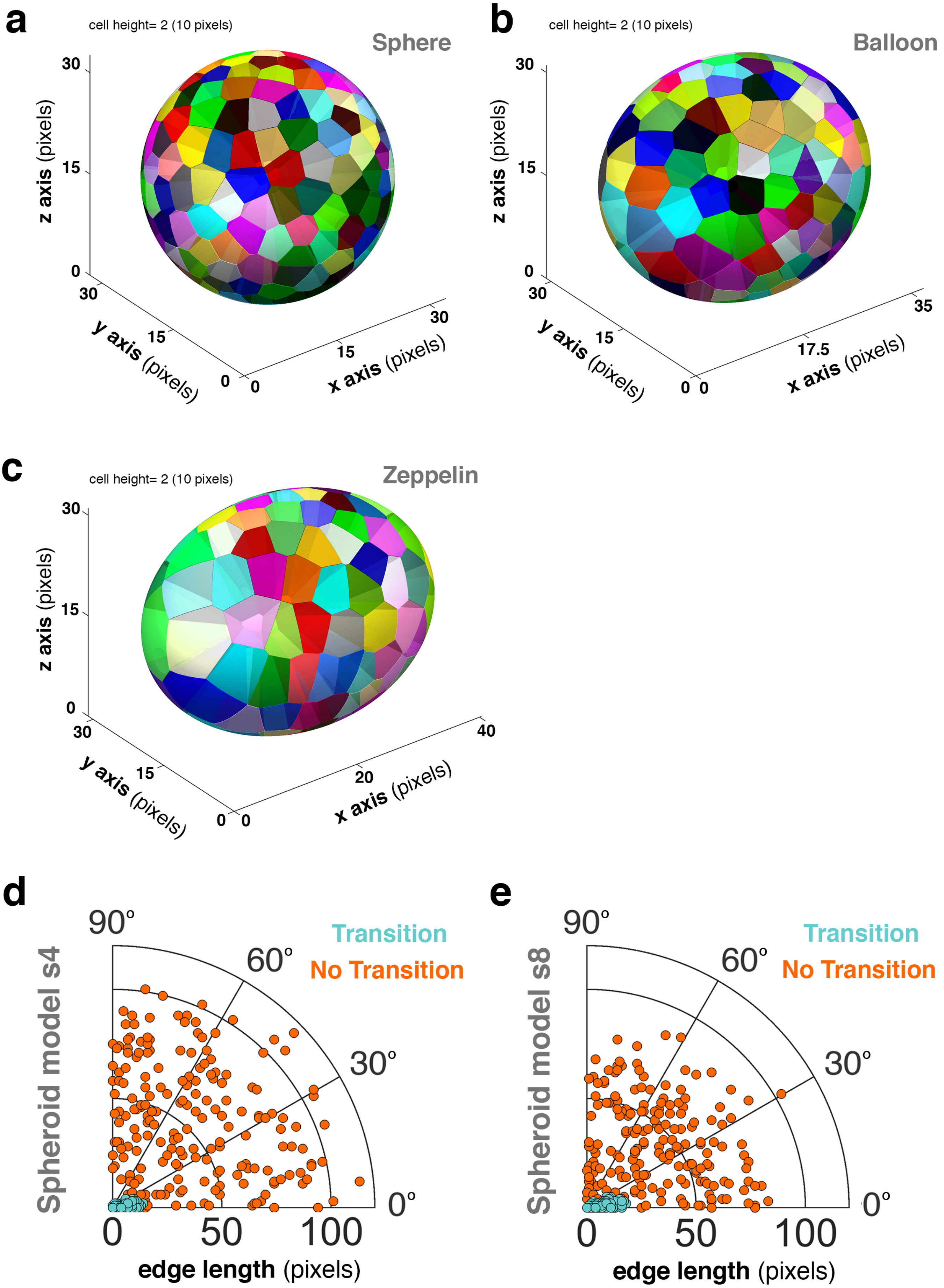

a
cell height= 2 (10 pixels)
Sphere
z axis (pixels)
y axis (pixels)
x axis (pixels)
30
15
0
b
cell height= 2 (10 pixels)
Balloon
z axis (pixels)
y axis (pixels)
x axis (pixels)
35
17.5
c
cell height= 2 (10 pixels)
Zeppelin
z axis (pixels)
y axis (pixels)
x axis (pixels)
40
20
d
Spheroid model s4
Transition
No Transition
90°
60°
30°
0°
0
50
100
edge length (pixels)
e
Spheroid model s8
Transition
No Transition
90°
60°
30°
0°
0
50
100
edge length (pixels)

Supplementary Figure 5. Scutoids appear in Voronoi spheroidal models. a-c) Voronoi spheroidal models with different elongations. The represented spheroids have the same Y and Z apical radius and the cell height is twice as much this value. The panel (**a**) represents a 'sphere' with its 3 axial-radii equals; (**b**) is a Voronoi spheroidal model with 'balloon' shape: X apical radius 1.5 times larger than the Y and Z apical radii; (**c**) shows a Voronoi spheroidal model with 'zeppelin' shape: X apical radius is two times larger than the Y and Z apical radii. See **Methods**. **d-e)** Polar scatter plots showing the length and the angle of the contacting edge in basal four-cell motifs from the Voronoi spheroid models s4 (**d**) and s8 (**e**) represented in **Fig. 5e** and **Fig. 5f** respectively. Light blue points stand for motifs that exchange neighbours; orange points stand for motifs that do not intercalate.

	angles ("Transition" vs "No transition")		length "Transition" vs "No transition")	
	h	p	h	p
**Salivary Gland**	1	4.98E-19	1	3.03E-21
**Tubular model - surface ratio 1/0.8**	1	2.26E-36	1	7.69E-62
**Tubular model - surface ratio 1/0.6**	1	1.30E-23	1	2.93E-39
**Tubular model - surface ratio 2**	1	6.93E-28	1	1.10E-17
**Tubular model - surface ratio 5**	1	5.36E-15	1	1.74E-06
**Embryo surface**	0	0.427	1	0.004
**Embryo fold**	1	3.19E-08	0	0.446
**Egg chamber Stage 4**	0	0.121	1	3.12E-14
**Egg chamber Stage 8**	1	0.012	1	2.22E-09
**Spheroid Model Stage 4**	1	1.06E-112	1	2.82E-263
**Spheroid Model Stage 8**	1	1.60E-67	1	2.42E-149
**Zebrafish**	0	0.582	1	0.000

h=1 reject the null hypothesis in with both samples belong to the same distribution

Supplementary Table 1: **Kolmogorov Smirnov test results comparing the distributions of edge angle and length in ‘transition’ and ‘no transition’ cases.** The analyses were performed over data obtained from measurements the Voronoi models and the actual tissues.

	mean % scutoids	std % scutoids	mean total cells in ROI	std total cells in ROI
**Sphere - cell height 0.5**	0.0%	0.0%	97.6	2.5
**Sphere - cell height 1**	0.0%	0.0%	97.6	2.5
**Sphere - cell height 2**	0.0%	0.0%	97.6	2.5
**Balloon - cell height 0.5**	21.7%	6.7%	77.0	1.8
**Balloon - cell height 1**	43.5%	7.7%	77.0	1.8
**Balloon - cell height 2**	61.6%	10.1%	77.0	1.8
**Zeppelin - cell height 0.5**	40.2%	9.3%	54.0	2.7
**Zeppelin - cell height 1**	68.7%	9.2%	54.0	2.7
**Zeppelin - cell height 2**	89.2%	5.1%	54.0	2.7
**Spheroid Model Stage 4 - cell height 0.2**	6.3%	3.4%	117.7	2.9
**Spheroid Model Stage 8 - cell height 0.14**	10.5%	3.5%	236.4	4.9

	Inner surface radii		
	**X**	**Y**	**Z**
**Sphere**	1	1	1
**Balloon**	1.5	1	1
**Zeppelin**	2	1	1
**Spheroid Model Stage 4**	1.2	1	1
**Spheroid Model Stage 8**	2.1	1	1

Supplementary Table 2: Percentage of scutoids measured in Voronoi spheroidal models. Data for the sphere, zeppelin, balloon, stage 4 and stage 8 models.
