# Supplementary material for: Scutoids are a geometrical solution to three-dimensional packing of epithelia
Source: Nat Commun. 2018 Jul 27;9:2960. doi: 10.1038/s41467-018-05376-1 (PMC6063940; doi:10.1038/s41467-018-05376-1)
Supplement: Supplementary file 3 — Description of Additional Supplementary Files [file 41467_2018_5376_MOESM3_ESM.pdf]

Description of Additional Supplementary Files

File Name: Supplementary Movie 1
Description: **Sequence of frames of a salivary gland processed stack.** The cells contours have been identified in every frame of the stack. Each cell has been tracked and labelled along the different confocal sections.

File Name: Supplementary Data 1
Description: **Frequency of apico-basal transitions for different surface ratios.** Percentage of cells with "transitions" and frequency of changes per cells in the Voronoi tubular model. Data are classified depending on the value of the surface ratio and the number of cells in the Voronoi tubular model (40, 80, 200, 400 and 800 cells) and collected from 20 different initial conditions.

File Name: Supplementary Data 2
Description: **Edge angles and edge length measurements in actual epithelia.** Data obtained from the four-cell motifs that have been analysed in *Drosophila* salivary glands and *Drosophila* embryo, *Drosophila* egg chamber and 50% epiboly Zebrafish embryo. Lengths and angles were always measured in the largest surface: the apical surface in the case of the *Drosophila* embryos and in 50% epiboly Zebrafish embryos; the basal surface in the case of the *Drosophila* salivary glands, the *Drosophila* egg chambers and *the Drosophila* embryo folds.

File Name: Supplementary Data 3
Description: **Line-tension minimization model input measurements.** Data from the *Drosophila* salivary gland, *Drosophila* egg chamber stage 4 and stage 8, and theirs corresponding Voronoi models.
